# Trends in Home Cooking among United States Adults from 2003 to 2023: Analysis of American Time Use Survey Food Preparation

**DOI:** 10.1016/j.cdnut.2025.107529

**Published:** 2025-08-23

**Authors:** Laina Ewoldt, Shu Wen Ng, Barry M Popkin, Lindsey S Taillie

**Affiliations:** 1 Department of Nutrition, Gillings School of Global Public Health, University of North Carolina, Chapel Hill, NC, United States; 2 Carolina Population Center, University of North Carolina, Chapel Hill, NC, United States

**Keywords:** cooking, food preparation, time use, sex, education

## Abstract

**Background:**

Home food preparation, “cooking,” can be an affordable method for improving diet quality and reducing intake of ultraprocessed foods, 2 important drivers of diet-related chronic diseases. Understanding current trends among United States adults can inform nutrition interventions promoting home cooking.

**Objective:**

The objective of this study was to determine the percentage of United States adults engaging in home cooking and the mean minutes per day spent cooking among those who cook.

**Methods:**

American Time Use Survey data were used to estimate the percentage of United States adults (*n* = 231,657) spending any time on home cooking and the mean daily cooking time among those cooking. Changes from 2003 to 2023 were assessed using linear regression and t-statistics. Subgroup analyses explored differences by sex and educational attainment.

**Results:**

From 2003 to 2023, percentage cooking increased among men [36% (SE 0.6%) to 52% (SE 0.9%) (*P* < 0.001)] and women [69% (SE 0.5%) to 72% (SE 0.9%) (*P* < 0.001)]. Mean time cooking among those who cook increased for men [45 min/d (SE 0.9) to 50 min/d (SE 1.2); *P* < 0.001], but not for women [71 min/d (SE 0.8) to 71 mi/d (SE 1.4); *P* = 0.869]. The largest increases in percentage cooking for men and women were among those with a college degree or higher [+18 percentage points (SE 1.7) men (*P* < 0.001); +7 percentage points (SE) women (*P* < 0.001)]. For time spent cooking, the largest increases were observed among women with less than a high school degree [+24 minutes/day (SE 9.9); *P* = 0.014] and among men for those with a college degree or higher [+11 minutes/day (SE 2.2); *P* < 0.001].

**Conclusions:**

The percentage of United States adults cooking increased since 2003, with larger increases among men. However, women are still the most likely to cook and spend the most time cooking, with large time increases among those with less than a high school education. Other increases were primarily observed in upper educated households.

## Introduction

Poor dietary quality, including high intakes of ultraprocessed food and food-away-from-home, is associated with an array of adverse health outcomes, including increased BMI, type 2 diabetes, and cardiovascular disease [[1], [2], [3]]. Home food preparation, “cooking,” offers an affordable strategy for reducing ultraprocessed food intake and away-from-home intake [4]. Indeed, several studies have found that increased home cooking is associated with increased intake of fruits and vegetables and overall diet quality [[4], [5], [6], [7]], whereas others have identified an association with lower adiposity and hazard of type 2 diabetes [6,8]. However, prior research has found that overall cooking times in the United States have decreased since the mid-20th century, posing questions about the feasibility of home cooking as a strategy for improving populations’ diets [9].

One important question is whether trends in time spent cooking have changed over recent years. A 2016 assessment of time spent cooking among United States adults indicated a slight uptick in home cooking compared with 2003 [10], and a study of French cooking habits during COVID-19 lockdowns reported increases in home cooking among 42% of participants [11]; however, it is unclear if changes in time usage have waned alongside other pandemic-associated changes. Additionally, observed trends have varied by sociodemographic characteristics. Increases observed in the United States were primarily driven by increases among men; however, women still performed the vast majority of cooking [10], reflective of global trends in the distribution of burden for cooking and other household activities [12]. Research has observed further variations by household income, as low-income individuals may face more barriers to home cooking in the form of limited food access or affordability, additional time requirements for budgeting grocery lists, lack of cooking equipment, or worry about wasting food when trying new recipes or ingredients [[13], [14], [15], [16], [17]]. These barriers likely contribute to observed trends of low-income households spending less time cooking and using more time-saving prepackaged or frozen products [18]. Despite the abundance of research investigating home cooking among low-income households, there remains a paucity of research understanding these same trends by education status. This research gap must be addressed to further alleviate health disparities, as lower educational status is associated with reduced diet quality [[19], [20], [21], [22]] and positively associated with chronic diseases such as cardiovascular disease and type 2 diabetes [23,24].

Understanding the percentage of the population cooking and the mean time spent preparing meals at home provides critical information for developing policies and programs promoting improved nutrition. Additionally, understanding baseline cooking statistics for the target population will inform realistic intervention development and goal setting. Therefore, the current study aimed to characterize current trends in home cooking among United States adults, which will help inform targets for nutrition interventions promoting home cooking. The study achieved this through 2 aims. First, the study explored the percentage of adults performing any cooking on an average day in the United States, both as a whole and stratified by sex and education. Second, the study quantified the mean minutes per day spent on cooking among those who spent any time cooking, both total and stratified by sex and education.

## Methods

### American Time Use Survey

The American Time Use Survey (ATUS) is a continuous federally administered nationally representative survey administered to civilian noninstitutionalized individuals aged 15 y or older who previously finished 8 Current Population Survey interviews [25]. To improve external reliability, some groups are oversampled, including Hispanic or non-Hispanic Black households and households with children. Given the continuous nature of the surveys, annually from January to December, interviewers collect data most days throughout the year (excluding government holidays and other closings).

The survey measures percent participation and mean hours in various activities through a computer-assisted telephone interview. During the interview, trained telephone interviewers conduct a 24 h recall from 04:00 the day before the interview to 04:00 the day of the interview. The time use section of the interview is open-ended, asking participants to walk the interviewer through each subsequent activity performed [“Yesterday, (previous day) at 4:00, what were you doing?/What did you do next?”], providing the start and stop time of each activity in addition to who the activity was completed with and where it occurred. Reported activities are all assigned a code from the activity classification system. Commonly reported activities, such as sleeping or preparing meals, are quickly assigned codes during the interview, whereas activities that do not fall within these categories are recorded verbatim and assigned a code afterward. Only primary activities are recorded, meaning that, for example, if a participant is passively cooking food in the oven while working on other household activities, cooking will be considered a secondary activity and will not be recorded. Demographic, household composition, and labor force participation information is sourced from participants’ final completed Current Population Survey interview, which is reviewed with participants during the ATUS interview and updated as needed. Additional modules, such as the Eating and Health Module (EHM), are administered in some years to capture other participant characteristics such as Supplemental Nutrition Assistance Program (SNAP) participation.

### Data extraction

Data was extracted from the IPUMS American Time Use Survey Extract Builder [26], and survey data was downloaded for all years ranging from 2003 to 2023 to allow for visualization of overall cooking trends within the period of interest. Data from 2020 is not representative of the entire year due to disruptions in data collection stemming from COVID-19, and therefore was not included when depicting yearly trends. The main analysis of cooking time focused on 2003 and 2023; however, 2006 and 2022 were used to capture SNAP participant time use, which was only collected during years that the EHM was administered. Separate probability and replicate weights were used for the primary time use module and the EHM.

### Cooking time

The cooking time variable used for this study was Household Activity 020200, food and drink preparation, presentation, and clean-up. The entire 24-h time frame was included. The selected variable is comprehensive of all aspects of cooking and includes activities such as pouring beverages, setting the table, cleaning the kitchen, defrosting food, and pumping breast milk. The variable does not include any work-related cooking activities, such as preparing or serving food as a chef or server. Activities involved in planning meals, such as shopping for food, are not considered in this estimate of cooking time.

The primary outcomes of interest were the percentage of respondents who reported any time cooking (food and drink preparation, presentation, and clean-up >0 min/d), and the mean time spent cooking among those reporting any time cooking.

### Covariates

Covariates were selected on the basis of their expected impact on time use or cooking-related attitudes and behaviors from past research [[27], [28], [29], [30]]. The included covariates include self-reported sex (male/female), age group (18–29, 30–44, 45–60, and 61+ y of age), children <18 y in the household (yes/no), living with a partner (yes/no), SNAP participation in the last 30 d (yes/no), employment status (full time at 35 h/wk or more, part time, and not in labor force), highest educational attainment (less than high school, high school, some college, and college or higher), and race/ethnicity (Hispanic, non-Hispanic Black, non-Hispanic Other, and non-Hispanic White). Other included individuals who reported >1 race.

### Exclusion criteria

Respondents were excluded from the analytical sample if they reported being on holiday during the interview, if they were aged <18 y of age, or if they reported an unrealistic amount of time spent cooking (>8.5 h/d).

### Statistical analysis plan

Descriptive statistics were analyzed to understand the demographics of the sample. For all analyses, the ATUS developed probability weights and replicate weights were applied to the study sample to make the sample nationally representative, accounting for oversampling and differential response rates.

Linear regression was used to conduct a test for trend in the percentage of United States adults cooking and the mean time spent cooking among those who cook across time, considering all years from 2003 to 2023 (excluding 2020). Changes from 2003 and 2023 were assessed using t-statistics derived from linear combinations of estimated means. Further subgroup analyses were conducted, stratifying by sex and education. Finally, sensitivity analyses were conducted for the overall trends from 2003 to 2023 that controlled for changes in population demographics across the years.

Statistical analysis was conducted using Stata version 17.0 [31].

## Results

The final unweighted sample included 231,657 participants from 2003 to 2023, with 19,533 respondents in 2003 and 8240 respondents in 2023 (Table 1). Compared with 2003, the weighted 2023 survey population had an increase in the percentage of older adults, higher educated respondents, and households without children, which reflects shifts in the United States population. The largest changes occurred in the college degree or higher educated group, increasing from 26% to 39%.

**TABLE 1:** Study population demographics in 2003 and 2023.

	2003		2023	
	*n* (%)	Weighted % (SE)	*n* (%)	Weighted % (SE)
Overall	19,533		8240	
Sex				
Male	8482 (43)	48 (0.1)	3851 (47)	49 (0.2)
Female	11,051 (57)	52 (0.1)	4389 (53)	51 (0.2)
Age group (y)				
18–29	2874 (15)	22 (0.1)	861 (10)	20 (0.2)
30–44	6641 (34)	30 (0.1)	1875 (23)	26 (0.1)
45–60	5683 (29)	28 (0.1)	1897 (23)	25 (0.2)
61+	4335 (22)	20 (0.1)	3607 (44)	29 (0.2)
Children <18 in household				
Yes	8622 (44)	39 (0.2)	2123 (26)	32 (0.2)
No	10,911 (56)	61 (0.2)	6117 (74)	68 (0.2)
Living with partner				
Yes	11,777 (60)	63 (0.4)	4831 (59)	59 (0.6)
No	7756 (40)	37 (0.4)	3409 (41)	41 (0.6)
Employment status				
Full time	10,333 (53)	53 (0.4)	3909 (47)	55 (0.6)
Part time	2534 (13)	14 (0.3)	936 (11)	12 (0.5)
Not in labor force	6666 (34)	33 (0.3)	3395 (41)	33 (0.5)
Race/ethnicity				
Hispanic	2143 (11)	12 (0.1)	1078 (13)	17 (0.2)
Non-Hispanic Black	2228 (11)	11 (0.1)	816 (10)	12 (0.2)
Non-Hispanic other	828 (4)	5 (0.2)	554 (7)	8 (0.4)
Non-Hispanic White	14,334 (73)	72 (0.2)	5792 (70)	63 (0.4)
Education level				
<High school	2648 (14)	15 (0.4)	480 (6)	8 (0.4)
High school	5719 (29)	33 (0.4)	1878 (23)	30 (0.5)
Some college	5447 (28)	26 (0.3)	2179 (26)	23 (0.5)
College and higher	5719 (29)	26 (0.3)	3703 (45)	39 (0.5)
SNAP^1^				
Yes	826 (7)	6 (0.3)	619 (8)	9 (0.4)
No	10,962 (93)	94 (0.3)	6908 (92)	91 (0.4)
Missing	161 (1)		304 (4)	

*n* = unweighted sample size.

1 SNAP comparison years are 2006 and 2022; Missing category includes those who refused to answer, responded that they were unsure, or those who did not complete the EHM.

**FIGURE 1:**
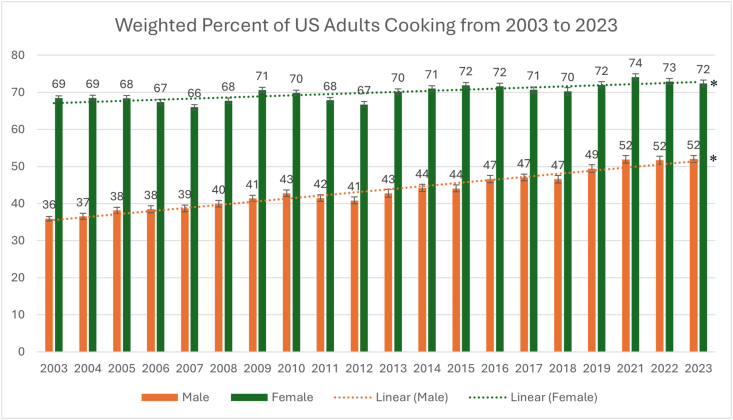
Weighted percent of United States adults cooking from 2003 to 2023 by sex. ∗P < 0.05 for linear regression test for trend between 2003 and 2023.

The percentage of women who cooked increased from 69% (SE 0.5%) in 2003 to 72% (SE 0.9%) in 2023 (*P* < 0.001), whereas the percentage of men who cooked increased from 36% (SE 0.6%) to 52% (SE 0.9%) in 2023 (*P* < 0.001). Visually assessing the overall trends for all included study years shows that the percentage of women cooking oscillated within an 8-percentage point range (66%–74%), whereas the percentage of men cooking steadily increased from 36% to 52% before stabilizing in 2021 (Figure 1). Linear regression shows a statistically significant mean 0.8 point (SE 0.0%) annual increase in the percent of males cooking (*P*-trend < 0.001) and a small but statistically significant 0.3 point (SE 0.0) annual increase in the percent of females cooking (*P*-trend < 0.001).

**FIGURE 2:**
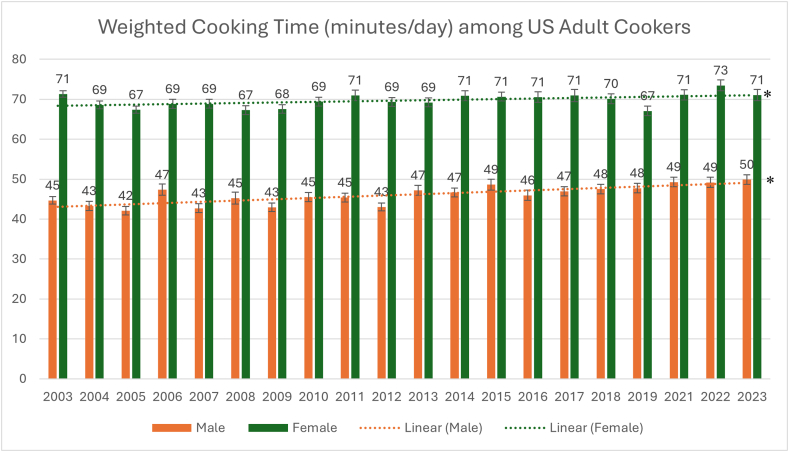
Mean per capita minutes per day spent cooking among United States cookers. ∗P < 0.05 for linear regression test for trend between 2003 and 2023.

The mean daily time spent cooking among women who cooked did not significantly change from 2003 to 2023 (71 min/d (SE 0.8)) to 71 min/d (SE 1.4); *P* = 0.869), whereas the mean daily time spent cooking among men who cooked increased from 45 min/d (SE 0.9) to 50 min/d (SE 1.2) in 2023 (*P* < 0.001). Among cookers, daily time spent cooking varied from 67 to 73 min/d for women between 2003 and 2023, and from 42 min/d to 50 min/d among men (Figure 2). Linear regression revealed a 0.3 min/d (SE 0.0) annual increase for men (*P*-trend < 0.001) and a 0.1 min/d (SE 0.0) annual increase for women (*P*-trend = 0.003). Regardless of meeting criteria for statistical significance, increases seen among both men and women signify minimal increase in daily time spent cooking among cookers.

Variations by sex and education level in the percentage spending any time cooking are displayed in Table 2. Statistically significant increases in the percentage cooking were seen among all categories of the displayed sociodemographic characteristics except for those with less than a high school education. However, a 4% (SE 3.3%) increase in the percentage cooking was seen alongside a large SE for the less than high school education category, so this group may have simply been insufficiently represented in the study to detect a small difference. Percentage cooking by additional sociodemographic characteristics can be found in Supplemental Table 1.

**TABLE 2:** Survey weighted percent of population cooking by select demographics.

	% Cooking (SE)			
	2003	2023	Difference^1^	*P*^2^
Overall (%)	53% (0.4%)	62% (0.6%)	10% (0.7%)	0.000
Sex				
Male	36% (0.6%)	52% (0.9%)	16% (1.1%)	0.000
Female	69% (0.5%)	72% (0.9%)	4% (0.9%)	0.000
Education level				
<High school	51% (1.2%)	55% (2.9%)	4% (3.3%)	0.219
High school	54% (0.7%)	62% (1.3%)	8% (1.5%)	0.000
Some college	52% (0.9%)	59% (1.3%)	7% (1.6%)	0.000
College and higher	53% (0.7%)	66% (1.0%)	13% (1.2%)	0.000

1 Calculated from unrounded values.

2 *P* value for t-test of difference between 2003–2023.

The largest increase in the percentage cooking by education level was seen in those with a college degree or higher (13% ± 1.2%). This increase over time also resulted in a higher percentage in this group cooking in 2023 than those with less than a high school degree [66% (SE 1.0%) compared with 55% (SE 2.9%)].

**FIGURE 3:**
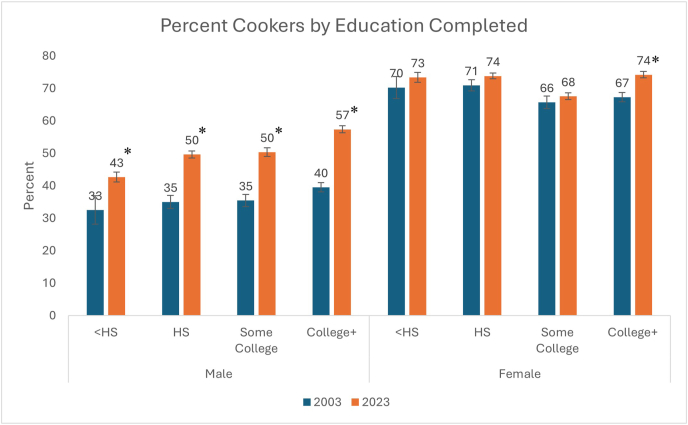
Bar charts of percentage cooking by sex and education level in 2003 and 2023. ∗ P < 0.05 for t-test of difference between 2003–2023; HS = high school, College+ = college degree or higher.

When stratifying by sex and education level (Figure 3), the largest increase in percentage cooking was seen among men with a college degree or higher [18 percentage points (SE 1.7%); *P* < 0.001] and the smallest increase observed among women with only some college [2 percentage points (SE 2.1%); *P* = 0.418]. Increases among men were statistically significant within all education strata; however, they were only significant among women with a college degree or higher.

Variations by sex and education level in the mean per capita time spent daily on cooking among those who spent any time cooking are displayed in Table 3.

**TABLE 3:** Survey weighted mean per capita daily time spent cooking among cookers by select demographics.

	Mean per capita daily time cooking among cookers (SE)^1^			
	2003	2023	Difference^2^	*P*^3^
Overall	63 (0.6)	62 (1.0)	0 (1.1)	0.849
Sex				
Male	45 (0.9)	50 (1.2)	5 (1.5)	0.000
Female	71 (0.8)	71 (1.4)	0 (1.6)	0.869
Education level				
<High school	77 (2.3)	85 (6.9)	8 (7.0)	0.227
High school	65 (1.4)	64 (2.0)	-2 (2.4)	0.439
Some college	59 (1.2)	58 (1.8)	0 (2.2)	0.922
College and higher	55 (1.1)	60 (1.1)	5 (1.5)	0.001

1 Minutes per person per day.

2 Calculated from unrounded values.

3 *P* value for t-test of difference between 2003–2023.

Mean per capita time spent cooking among cookers remained unchanged in most groups from 2003 to 2023; however, a 5 min/d (SE 1.5) increase (*P* < 0.001) was seen for those with a college degree or higher. Despite observed within-category increases over time, cookers with less than a high school education still cook more than those with a college degree in 2023 [85 min/d (SE 6.9) compared with 60 min/d (SE 1.1)]. Variation in mean per capita time spent daily on cooking among cookers by additional sociodemographic characteristics can be found in Supplemental Table 2.

**FIGURE 4:**
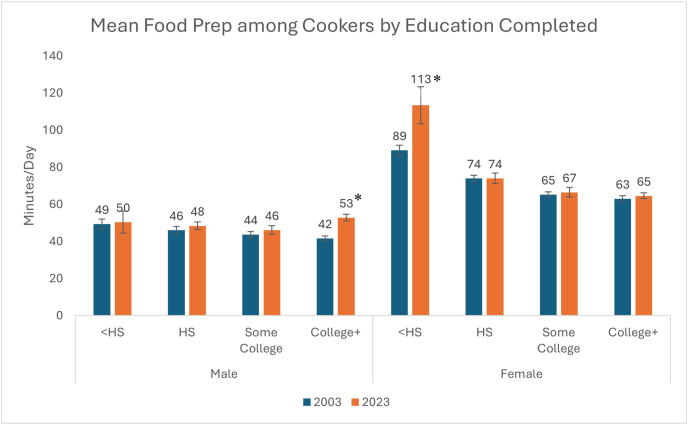
Bar charts of mean daily time cooking by sex and education level in 2003 and 2023 among cookers. ∗P < 0.05 for t-test of difference between 2003–2023; HS = high school, College+ = college degree or higher.

When stratifying by sex and education level (Figure 4), significant increases in time spent cooking by cookers were seen among women with less than a high school degree [24 min/d (SE 9.9); *P* = 0.014] and men with a college degree or higher [11 min/d (SE 2.2); *P* < 0.001].

### Sensitivity analysis

Sensitivity analyses suggested minimal impact of changes in population demographics on the observed trends from 2003 to 2023. The magnitude and significance of the observed trends for the percent of males and females cooking remained unchanged when adjusting for demographic changes over time (Supplemental Table 3). For daily cooking time among those cooking, the statistical significance of the observed trends remained unchanged; however, there was a slight increase in the magnitude of the increase observed for women [0.2 min/d annual increase (SE 0.05); *P*-trend < 0.001] (Supplemental Table 3).

## Discussion

The results of this study show that the percentage of United States adults cooking has increased since 2003; however, the overall mean time spent cooking among cookers has remained relatively stable. This indicates that, on average, more people are cooking; however, they are not dedicating more daily time to this activity. Despite larger increases observed among men, at all included time points, a higher percentage of women than men cooked, and more time was spent cooking among women who cook than among men. These results show a continuation of the trends observed in 2016, where the percentage of cooking increased overall, with a larger increase seen among men. In this 2016 study, 46% of men and 70% of women reported spending any time cooking [10], compared with the current study, which showed further increases, to 52% of men and 72% of women reporting any time spent cooking in the 2023 ATUS survey. The smaller increase in the percentage of women cooking may indicate a topping-off effect compared with men, who had a lower percentage cooking initially and therefore more opportunity for increased engagement with this activity.

This study also highlighted greater increases in the percentage cooking and per capita time spent cooking among upper educated adults. These findings agree with prior research in the United States and Denmark that identified a positive relationship between education and home cooking [10,32,33]; however, they conflict with global trends, which show a negative association between cooking frequency and education [12]. Increases observed in our study may be partly explained by those with higher educational attainment, considering health as a more influential driver when making decisions about food [34]. When stratifying by sex and education, the largest increase in percentage cooking was seen among those with a college degree or higher for both men and women. When considering time spent cooking, the college or higher educated group had the greatest increase in time spent cooking per cooker among men, whereas for women, the largest increase was seen among those with less than a high school education. This trend could indicate that in households with lower education, women perform more of the household cooking, which aligns with prior findings that contribution to household income has a greater impact on time spent on household tasks for women than for men [35]. The large increase in time spent cooking by women with less than a high school education may also indicate a lower reliance on convenience items such as precut produce or premeasured meal kits, which increase efficiency at the expense of higher food costs.

The findings of this study can inform future nutrition interventions focused on home cooking as a means to improve diet quality. The increases already observed in the percentage of United States adults cooking are encouraging; however, the percentage cooking remains low among men, with fewer than 60% reporting any cooking across education strata. These findings indicate that future interventions may benefit from targeting men, particularly those with lower educational attainment. Interventions aimed at involving children in household cooking should also encourage families to involve boys and girls equally in order to ensure that both learn the necessary cooking skills to support themselves later in life [36,37]. Cooking education for all sexes in schools and other community settings has the potential to improve food-related beliefs, knowledge, and behaviors during their formative years [38]. Prior school-based cooking interventions have seen improvements in fruit and vegetable preferences for both boys and girls involved in the programs [[39], [40], [41]], with one study seeing the largest improvement among students with no prior cooking experience, 61% of whom were boys [39]. Given that time is often considered a top barrier to home cooking [17], the findings related to the mean per capita time spent on cooking can inform cooking interventions in their development of recipes and plans cognizant of realistic time requirements.

Although women with less than a high school education had the highest mean time spent cooking in the current study, many living in low-income households could still reap benefits from home cooking interventions. Time scarcity, shelf-stability, and acceptability by household members can increase the intake of ultraprocessed foods, so attempts to limit their intake may need to promote desirable alternatives that do not drastically increase the time or financial burden of meal planning, shopping, or preparation [42,43]. Further, less developed cooking skills may increase cooking inefficiency and consequently time scarcity, which may partly explain why prior studies observed an association between cooking skills and ultraprocessed food intake [44,45], as many individuals use ultraprocessed foods to reduce cooking time. These factors can create especially difficult barriers for low-income individuals who face additional decisions throughout this process while trying to stretch limited food budgets. Programs such as SNAP-Ed and the Expanded Food and Nutrition Education Program have shown success addressing these needs through helping low-income individuals develop the skills and self-efficacy to plan and prepare budget-friendly, nutritious meals [46,47]; however, further work is needed to extend participant benefits over a longer follow-up period [46].

Readers should interpret these study findings within the context of wider cooking trends. Because ATUS data only records primary cooking activities and provides limited details about cooking methods, it alone is insufficient for understanding drivers of change in the percentage of adults cooking and time spent cooking. Popularization of cooking tools such as pressure cookers reduced the passive time required to cook meals, which may have reduced the perceived barriers to cooking, thereby increasing the percentage of United States adults cooking. Further, viral recipes for pressure cookers or one-pot/pan recipes in recent years often emphasized low active cooking time requirements, which could reduce cooking time observed in ATUS data without impacting the quantity or frequency of meals prepared within the home. Additionally, the survey data used for the current study do not include dietary data, limiting inferences about the effect of observed changes in cooking participation on diet quality. Although percentage currently cooking and mean time spent cooking still provide useful statistics when developing interventions, understanding current trends driving cooking participation will significantly strengthen program development within home cooking interventions. Future research would benefit from developing and utilizing tools that collect data on cooking participation alongside data on cooking equipment and techniques used, and diet quality [48].

This research had several notable strengths. First, the 24-h recall format decreases the chances that respondents altered their behaviors during the study day due to social desirability bias. Second, the usage of ATUS study weights increases the external validity of the study to the general United States population, while also reducing possible bias from variability in the sampling or response rate by sociodemographic characteristics. Finally, the large sample size provided sufficient power to precisely detect differences across study years. Despite the study’s strengths, a few weaknesses must still be considered. A 24 h recall was used to measure time use, and although they can accurately capture average time use in a population, the results do not necessarily reflect usual time use among individuals. As with all surveys dependent on participant recall, some measurement error could have been introduced by participants forgetting some activities or the time spent on them. This would likely impact the estimates of mean time spent cooking, but not the percentage cooking, as people are unlikely to forget every instance of cooking from the prior day. Measurement error may also be present from the incorrect categorization of time use activities as a different household task. Additionally, cooking time may be underestimated due to cooking as a secondary activity not being recorded. Across these various potential sources of measurement error, insufficient information is available to understand if the error systematically varies by subpopulations. Finally, the analysis does not consider the time burden of planning meals and shopping for food, or all household dynamics and the interactions between them that may impact time usage.

In conclusion, the percentage of adults cooking in the United States has increased since 2003, with larger changes in men than women, though a greater percentage of women still cook in 2023. Notably, increases in percent cooking predominantly occurred among upper educated households, which may exacerbate dietary inequalities given that cooking is associated with better diet quality [5,7]. Conversely, women with less than a high school education had the largest increase in mean time spent cooking among cookers compared to all other groups. Interventions targeting cooking education, skills development, and self-efficacy may be useful for increasing home cooking participation while also reducing barriers for those cooking.

## Author contributions

The authors’ responsibilities were as follows – LE, LST: designed research; LE: performed statistical analysis; LE: wrote the initial draft of the paper; SWN, BMP, LST: reviewed and edited the paper; LST: had primary responsibility for final content; and all authors: read and approved the final manuscript.

## Data Availability

ATUS data described in the manuscript and codebook are publicly available online at https://www.atusdata.org/atus/. Analytic code will be made available upon request.

## Funding

The authors reported no funding received for this study.

## Conflict of Interests

The authors report no conflicts of interest.
