# Supplementary material for: Trends in Home Cooking among United States Adults from 2003 to 2023: Analysis of American Time Use Survey Food Preparation
Source: Curr Dev Nutr. 2025 Aug 23;9(9):107529. doi: 10.1016/j.cdnut.2025.107529 (PMC12512152; doi:10.1016/j.cdnut.2025.107529)
Supplement: multimedia component 1 [file mmc1.docx]

Cooking Trends by Additional Sociodemographic Characteristics

Table S1: Survey weighted percent of population cooking by select demographics

|  | **% Cooking (SE)** | | | |
| --- | --- | --- | --- | --- |
|  | 2003 | 2023 | Difference* | p-value^†^ |
| Overall | 53% (0.4%) | 62% (0.6%) | 10% (0.7%) | 0.000 |
| Sex |  |  |  |  |
| *Male* | 36% (0.6%) | 52% (0.9%) | 16% (1.1%) | 0.000 |
| *Female* | 69% (0.5%) | 72% (0.9%) | 4% (0.9%) | 0.000 |
| Age Group |  |  |  |  |
| *18-29* | 39% (1.0%) | 54% (2.2%) | 15% (2.2%) | 0.000 |
| *30-44* | 55% (0.6%) | 64% (1.4%) | 8% (1.6%) | 0.000 |
| *45-60* | 55% (0.7%) | 62% (1.4%) | 7% (1.5%) | 0.000 |
| *61+* | 60% (0.8%) | 67% (0.8%) | 7% (1.2%) | 0.000 |
| Children <18 in household |  |  |  |  |
| *Yes* | 56% (0.5%) | 65% (1.2%) | 11% (0.9%) | 0.000 |
| *No* | 51% (0.6%) | 61% (0.8%) | 9% (1.2%) | 0.000 |
| Living with Partner |  |  |  |  |
| *Yes* | 56% (0.5%) | 65% (0.8%) | 9% (0.9%) | 0.000 |
| *No* | 47% (0.8%) | 59% (1.2%) | 12% (1.3%) | 0.000 |
| Employment Status |  |  |  |  |
| *Full Time* | 45% (0.7%) | 58% (1.0%) | 13% (1.1%) | 0.000 |
| *Part Time* | 56% (1.2%) | 65% (2.2%) | 9% (2.6%) | 0.000 |
| *Not in Labor Force* | 64% (0.8%) | 69% (1.0%) | 5% (1.3%) | 0.000 |
| Race/Ethnicity |  |  |  |  |
| *Hispanic* | 52% (1.1%) | 56% (2.0%) | 4% (2.2%) | 0.055 |
| *Non-Hispanic Black* | 49% (1.5%) | 56% (2.1%) | 7% (2.3%) | 0.003 |
| *Non-Hispanic Other* | 49% (2.4%) | 65% (2.6%) | 16% (3.4%) | 0.000 |
| *Non-Hispanic White* | 54% (0.5%) | 65% (0.8%) | 11% (0.9%) | 0.000 |
| Education Level |  |  |  |  |
| *<High School* | 51% (1.2%) | 55% (2.9%) | 4% (3.3%) | 0.219 |
| *High School* | 54% (0.7%) | 62% (1.3%) | 8% (1.5%) | 0.000 |
| *Some College* | 52% (0.9%) | 59% (1.3%) | 7% (1.6%) | 0.000 |
| *College and Higher* | 53% (0.7%) | 66% (1.0%) | 13% (1.2%) | 0.000 |
| SNAP‡ |  |  |  |  |
| *Yes* | 66% (2.1%) | 65% (2.6%) | -2% (3.2%) | 0.564 |
| *No* | 53% (0.6%) | 63% (0.8%) | 10% (1.0%) | 0.000 |
| * Calculated from unrounded values  † p-value for t-test of difference between 2003-2023  ‡ SNAP comparison years are 2006 and 2022 | | | | |

Table S2: Survey weighted mean per capita time spent cooking among those cooking by select demographics

|  | **Mean per capita time cooking among those cooking (SE)*** | | | |
| --- | --- | --- | --- | --- |
|  | 2003 | 2023 | Difference† | p-value‡ |
| Overall | 63 (0.6) | 62 (1.0) | 0 (1.1) | 0.849 |
| Sex |  |  |  |  |
| *Male* | 45 (0.9) | 50 (1.2) | 5 (1.5) | 0.000 |
| *Female* | 71 (0.8) | 71 (1.4) | 0 (1.6) | 0.869 |
| Age Group |  |  |  |  |
| *18-29* | 54 (1.7) | 48 (2.2) | -5 (2.9) | 0.068 |
| *30-44* | 64 (1.0) | 63 (2.0) | -1 (2.2) | 0.593 |
| *45-60* | 59 (1.2) | 68 (2.3) | 9 (2.5) | 0.000 |
| *61+* | 71 (1.5) | 65 (1.4) | -6 (2.2) | 0.013 |
| Children <18 in household |  |  |  |  |
| *Yes* | 68 (1.0) | 68 (2.1) | 1 (2.2) | 0.782 |
| *No* | 59 (0.9) | 59 (1.1) | 1 (1.5) | 0.706 |
| Living with Partner |  |  |  |  |
| *Yes* | 67 (0.8) | 67 (1.3) | 0 (0.0) | 0.972 |
| *No* | 53 (1.1) | 55 (1.4) | 2 (1.8) | 0.328 |
| Employment Status |  |  |  |  |
| *Full Time* | 50 (0.7) | 53 (1.1) | 3 (1.3) | 0.055 |
| *Part Time* | 62 (1.7) | 65 (2.9) | 3 (3.3) | 0.316 |
| *Not in Labor Force* | 77 (1.3) | 75 (1.8) | -2 (2.3) | 0.403 |
| Race/Ethnicity |  |  |  |  |
| *Hispanic* | 81 (2.4) | 80 (3.9) | -1 (4.6) | 0.838 |
| *Non-Hispanic Black* | 62 (2.0) | 64 (3.2) | 2 (3.7) | 0.673 |
| *Non-Hispanic Other* | 67 (3.5) | 75 (4.0) | 8 (5.5) | 0.137 |
| *Non-Hispanic White* | 59 (0.7) | 56 (0.9) | -3 (1.1) | 0.008 |
| Education Level |  |  |  |  |
| *<High School* | 77 (2.3) | 85 (6.9) | 8 (7.0) | 0.227 |
| *High School* | 65 (1.4) | 64 (2.0) | -2 (2.4) | 0.439 |
| *Some College* | 59 (1.2) | 58 (1.8) | 0 (2.2) | 0.922 |
| *College and Higher* | 55 (1.1) | 60 (1.1) | 5 (1.5) | 0.001 |
| SNAP§ |  |  |  |  |
| *Yes* | 77 (3.2) | 80 (4.2) | 3 (5.1) | 0.619 |
| *No* | 60 (0.9) | 62 (1.0) | 2 (1.4) | 0.145 |
| * Minutes per person per day  † Calculated from unrounded values  ‡ p-value for t-test of difference between 2003-2023  § SNAP comparison years are 2006 and 2022 | | | | |

Weighted Trends Further Adjusted for Demographic Shifts Across Years

Table S3: Weighted and further adjusted linear regression results of annual changes in percent of US adults cooking and time spent cooking among those cooking from 2003 to 2023

|  | Coefficient | Standard Error | p-value |
| --- | --- | --- | --- |
| **Annual change in percent cooking (%)** |  |  |  |
| *Male* | 0.8 | 0.03 | <0.001 |
| *Female* | 0.3 | 0.03 | <0.001 |
| **Annual change in mean per capita time cooking among those cooking (minutes/day)** |  |  |  |
| *Male* | 0.3 | 0.05 | <0.001 |
| *Female* | 0.2 | 0.05 | <0.001 |
| ATUS survey weights applied to adjust results to be nationally representative for a given year in addition to controlling for age group, children <18 in household, partner present in household, employment status, race/ethnicity, and educational attainment to adjust for potential demographic changes across years. | | | |
